# Oxytocin normalizes the implicit processing of fearful faces in psychopathy: a randomized crossover study using fMRI

**DOI:** 10.1038/s44220-023-00067-3

**Published:** 2023-05-25

**Authors:** John Tully, Arjun Sethi, Julia Griem, Yannis Paloyelis, Michael C. Craig, Steven C. R. Williams, Declan Murphy, Robert James Blair, Nigel Blackwood

**Affiliations:** 1 Department of Forensic and Neurodevelopmental Sciences, Institute of Psychiatry, Psychology and Neuroscience, Kings College London, London, UK; 2 Academic Unit of Mental Health and Clinical Neurosciences, School of Medicine, University of Nottingham, Jubilee Campus, Nottingham, UK; 3 Centre for Neuroimaging Sciences, Institute of Psychiatry, Psychology and Neuroscience, Kings College London, London, UK; 4 Child and Adolescent Mental Health Centre, Mental Health Services, Capital Region of Denmark, Copenhagen, Denmark

**Keywords:** Personality, Translational research

## Abstract

Adults with antisocial personality disorder with (ASPD + P) and without (ASPD – P) psychopathy commit the majority of violent crimes. Empathic processing abnormalities are particularly prominent in psychopathy, but effective pharmacological interventions have yet to be identified. Oxytocin modulates neural responses to fearful expressions in healthy populations. The current study investigates its effects in violent antisocial men. In a placebo-controlled, randomized crossover design, 34 violent offenders (19 ASPD + P; 15 ASPD – P) and 24 healthy non-offenders received 40 IU intranasal oxytocin or placebo and then completed an fMRI morphed faces task examining the implicit processing of fearful facial expressions. Increasing intensity of fearful facial expressions failed to appropriately modulate activity in the bilateral mid-cingulate cortex in violent offenders with ASPD + P, compared with those with ASPD – P. Oxytocin abolished these group differences. This represents evidence of neurochemical modulation of the empathic processing of others’ distress in psychopathy.

## Abstract (web summary)

Using a placebo-controlled, randomized crossover design, authors demonstrate that intranasal oxytocin modulated the processing of fearful facial expressions during an fMRI task in adults with antisocial personality disorder with psychopathy.

## Main

A small group of men engage in a life-course-persistent pattern of antisocial behavior^1^. These men are disproportionally responsible for violent crimes^2^, resulting in considerable personal and societal costs^3^. They meet diagnostic criteria for conduct disorder in childhood and antisocial personality disorder (ASPD) in adulthood. However, there is considerable heterogeneity within this group. Approximately one-third of men with ASPD meet additional diagnostic criteria for psychopathy (ASPD + P)^4^. They exhibit callous-unemotional traits in childhood^5^, begin offending at earlier ages and engage in a broader range and greater density of offending behaviors^6^ than those without psychopathy (ASPD – P). Importantly, they also respond less well to psychosocial treatment programmes^7^. Clinical guidelines suggest that currently available evidence does not support the use of pharmacological interventions for the treatment of antisocial personality disorder or its associated behaviors of aggression, anger and impulsivity^8^.

Abnormalities in reinforcement-based decision making and emotional (particularly empathic) responsiveness may help to explain the behaviors of these violent offenders. Decision-making abnormalities are observed in antisocial men with and without psychopathy^9^ when they undertake tasks in which they must learn which responses to make to gain a reward or to avoid punishment. There are differences in the neural response to unanticipated punishment between those with and without psychopathy^10^. Such impairments may underpin reduced reinforcement sensitivity, resulting in impulsivity, frustration-induced reactive aggression and recidivism^11^. By contrast, deficits in different components of empathic processing appear to be relatively specific to antisocial men with psychopathy. Behaviorally, individuals with psychopathy demonstrate impairments when explicitly asked to emotionally ‘label’ static two-dimensional images of a range of facial emotions^12^, most markedly for fear and sadness^12,13^. Functional magnetic resonance imaging (fMRI) work indicates altered neural responses to empathy-inducing pictures of physically painful situations in individuals with psychopathy^14^. A reduced ability to recognize and respond to another’s fear, pain and distress may be related to the use of goal-directed instrumental aggression that is particularly prevalent in individuals with psychopathy because the individual is less concerned by the distress of others and less fearful of punishment^15^.

However, the exploration of a key aspect of empathic processing—implicit neural responses to others’ facial expressions of fear—in violent antisocial men with ASPD, with and without psychopathy, has so far been limited. In healthy individuals, partially separable neural systems are involved in the processing of specific emotions, with prominent roles for the amygdala, insula, and anterior and mid-cingulate cortex in processing fearful expressions^13,16,17^ (but see also ref. ^18^). Studies in antisocial youth suggest reduced amygdala activity in response to fearful faces in children with high levels of callous-unemotional traits (the developmental precursor of ASPD + P)^19^ but increased amygdala activity in those with low levels of callous-unemotional traits (the developmental precursor of ASPD – P)^20^, compared with typically developing children. In adults, only two previous studies have been conducted to explore the implicit neural processing of fearful facial expressions in violent antisocial men categorized as ASPD +/– P. In a small pilot study^21^, men with ASPD + P (compared with healthy non-offenders) showed significantly reduced activation in the core face-processing network^16^ in response to fearful facial expressions at ‘low’ and ‘prototypical’ intensities. In a larger study^22^, men with ASPD + P (compared with men with ASPD – P) showed reduced activation in the core face-processing network and associated emotional and motivational processing regions (orbitofrontal cortex and ventromedial prefrontal cortex), but increased dorsal insula responses, when passively viewing dynamic facial expressions of fear. However, this study lacked a non-offender control group. Two other studies conducted in samples of violent male offenders assessed the dimensional impact of psychopathic traits on implicit neural responses to emotional faces^23,24^. However, in one, not all violent offenders had ASPD^23^, while in the other, participants were not assessed for presence of ASPD^24^.

Furthermore, no study to our knowledge has investigated whether group differences in brain activation can be modified by pharmacological agents. One potential agent is oxytocin, a neuropeptide central to the regulation of complex social behaviors. Oxytocin has a key role in social functions such as emotion recognition^25^, binding to receptors in social brain regions reported as functionally abnormal in ASPD (such as the amygdala and cingulate cortex^26^). In healthy individuals, oxytocin enhances the explicit emotional recognition of fearful faces^27^ and significantly impacts activity within fearful facial-processing regions, including the amygdala, insula and anterior cingulate cortex^28^. In antisocial adults, a small behavioral study^29^ has suggested that a single dose of 24 IU intranasal oxytocin can improve fearful expression recognition, at least in the short term. However, no previous work has examined the neural basis of this potential effect.

Hence, we carried out a double-blind, placebo-controlled, randomized crossover study in male violent offenders with ASPD + P and ASPD – P and healthy non-offenders to explore the impact of oxytocin on brain functional differences when implicitly processing others’ distress in the form of fearful facial expressions at varying intensities. Considering previous studies investigating processing of facial expressions of fear and studies investigating regional effects of oxytocin, we hypothesized that (1) violent offenders with ASPD + P would show reduced modulation by fearful expression intensity within amygdala, anterior insula and bilateral anterior/mid-cingulate cortices compared with both violent offenders with ASPD – P and healthy non-offenders (NO) under placebo and (2) intranasal oxytocin administration would reduce group differences in neural responding to fearful expression intensity within these regions.

## Results

### Behavioral data

Across the whole sample, participants successfully performed the gender rating task (mean accuracy 96.7% (s.d. = 1.0), mean response latency = 938.8 milliseconds (s.d. = 183.3)). For accuracy, there were no significant effects of group (NO, ASPD – P or ASPD + P; *η*_P_^2^ = 0.071, *F*_2,55_ = 2.114, *P* = 0.13), condition (placebo or oxytocin, *η*_P_^2^ = 0.017, *F*_1,42_ = 0.553, *P* = 0.333) or intensity of emotion (40%, 60%, 80% or 100%; *η*_P_^2^ = 0.33, *F*_1,42_ = 1.899, *P* = 0.174). For response latency, there were no significant effects of group (*η*_P_^2^ = 0.41, *F*_1,42_ = 1.174, *P* = 0.317), condition (placebo or oxytocin, *η*_P_^2^ = <0.001, *F*_1,42_ = 0.001, *P* = 0.981) or intensity of emotion (*η*_P_^2^ = 0.38, *F*_1,42_ = 2.17, *P* = 0.146). No group-by-intensity interactions for accuracy or response latency were observed.

### fMRI results

#### Parametric modulation of neural responses by emotion intensity

The main 3dMVM (analysis of variance-style computations) analysis revealed significant activation of the right middle occipital gyrus, involving the primary visual cortex, extending into the right fusiform gyrus; the left middle occipital gyrus, extending into the left fusiform gyrus; and a separate region within the left fusiform gyrus, associated with modulation by fearful expressions (further details in Supplementary Information section 6).

#### Group differences in responses to modulated fearful expressions

**Fig. 1:**
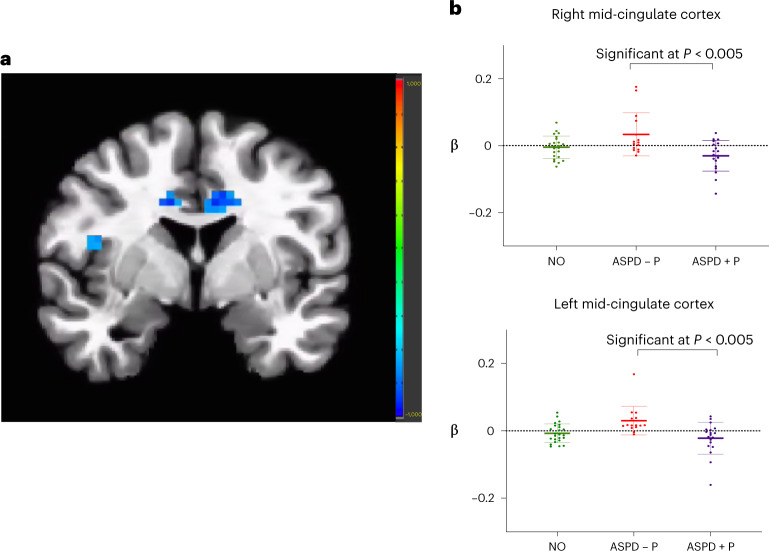
Group differences in placebo condition. **a**, Reduced modulation by fearful intensity in bilateral mid-cingulate cortex and right anterior insula in violent offenders with ASPD + P (*n* = 19) compared with violent offenders with ASPD – P (*n* = 15), placebo condition, *P* = 0.005. Color bar represents *t* statistic. **b**, Individual beta values for fear processing (modulated fear regressor) in bilateral mid-cingulate cortex, placebo condition, *P* = 0.005. Individual participants’ data plotted as dots. Means are indicated by horizontal bars. Error bars represent standard deviations. Findings in insula did not survive multiple comparison corrections. For NO, *n* = 24.
- Violent offenders with ASPD + P showed significant increases in modulation by fearful expression intensity in bilateral mid-cingulate cortex and left anterior insula under the oxytocin relative to the placebo condition (Fig. 2a,b).

In the three-group 3dMVM regions of interest (ROI) analyses for fearful expressions, there was an overall effect of group in the right and left mid-cingulate cortex (Table 1). Exploratory post hoc between-group analyses revealed four key findings:

- Violent offenders with ASPD + P showed reduced modulation of blood-oxygen-level-dependent (BOLD) responding by fearful expression intensity within bilateral mid-cingulate and right anterior insula compared with the group of violent offenders with ASPD – P under placebo conditions (Fig. 1a,b). The right anterior insula finding did not survive strict correction for multiple comparisons.

**Fig. 2:**
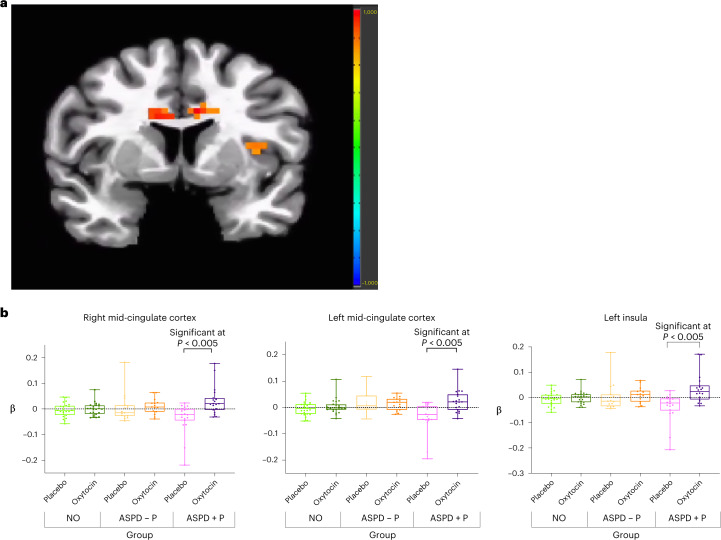
Effect of oxytocin in ASPD + P. **a**, Increased modulation by fearful intensity in bilateral mid-cingulate cortex and left insula in oxytocin relative to placebo condition in violent offenders with ASPD+P (*n* = 19), *P* = 0.005. Color bar represents *t* statistic. **b**, Individual beta values for fear processing (modulated fear regressor) for contrast oxytocin > placebo in bilateral mid-cingulate cortex and left insula in violent offenders with ASPD + P (*n* = 19). Individual participants’ data plotted as dots. Bounds of upper whiskers represent maxima; bounds of lower whiskers represent minima; center box represents interquartile range, with mean represented by middle horizontal lines.
- There were thus no group differences between ASPD + P and ASPD– P in the oxytocin condition; that is, differences under placebo were abolished under oxytocin. This was supported by a statistically significant group (ASPD – P, ASPD + P) by condition (placebo, oxytocin) interaction effect in left mid-cingulate cortex (Supplementary Figs. 3 and 4).
- There were no significant main effects or between- or within-group effects findings in amygdala in either placebo or oxytocin condition.

**Table 1:** Significant BOLD responses to modulated fearful expressions (covaried for active substance misuse)

Region	BA	Voxels	*X* (left to right)	*Y* (posterior to anterior)	*Z* (inferior to superior)	Statistic^a^	*P*
**Overall group effect**							
Right mid-cingulate gyrus	24	29	+13.5	−4.5	−32.5	9.464 (*F*)	0.0003
Left mid-cingulate gyrus	23/24	28	−10.5	−10.5	−35.5	9.135 (*F*)	0.0003
**ASPD** + **P** < **ASPD** **–** **P (placebo condition)**							
Left mid-cingulate gyrus	23/24	109	−7.5	−13.5	−35.5	3.95	0.0002
Right mid-cingulate gyrus	24	92	+10.5	−4.5	−32.5	4.115	0.0001
Right anterior insula^b^	13	6	+40.5	−4.5	−17.5	3.045	0.003
**Effect of oxytocin in ASPD** + **P (oxytocin** > **placebo)**							
Right mid-cingulate gyrus	24	173	+10.5	+1.5	−26.5	4.033	0.0001
Left mid-cingulate gyrus	24	119	−10.5	+10.5	−29.5	4.074	0.0001
Left anterior insula	13	15	−34.5	+4.5	−14.5	3.498	0.0009
**Group (ASPD** + **P versus ASPD** **–** **P)** **×** **condition (placebo versus oxytocin) interaction effect**							
Left mid-cingulate gyrus	23/24	16	−7.5	−13.5	−32.5	3.21	0.0022

Hypothesized regions first, then ordered by cluster size. Significance threshold set at *P* ≤ 0.005; all findings significant after cluster-wise correction for multiple comparisons unless noted otherwise. BA, Brodmann area.

^a^Statistic refers to *t*-tests unless stated otherwise.

^b^Findings not significant after cluster-wise correction for multiple comparisons.

## Discussion

We investigated the neural basis of implicit fearful facial emotion processing in violent offenders with antisocial personality disorder with and without psychopathy and the effect of intranasal oxytocin on brain functional differences. Offenders with ASPD + P displayed reduced modulation by fearful expression intensity in the anterior insula and mid-cingulate cortex (but not the amygdala) compared with offenders with ASPD – P. Oxytocin abolished differences in fear-associated activity within bilateral mid-cingulate cortex for the offenders with ASPD + P.

The identification of reduced anterior insula and mid-cingulate cortex reactivity to implicit processing of facial expressions of fear in ASPD + P relative to ASPD – P is broadly consistent with the previous literature^19–22^. The anterior insula is critical in representing the salience of stimuli^30^. It generates an integrated awareness of one’s cognitive, affective and physical state that becomes re-represented in the anterior cingulate cortex to facilitate homeostatic autonomic and behavioral responses^31^. The mid-cingulate cortex is a key part of reactive fear circuitry, helping to inform rapid decisions of escape from predators, which may be signaled by the fearful face of a conspecific^32^. It appears to coordinate emotional responses and motor actions according to learned values, particularly when a predatory threat is near^32^. Especially robust links have been demonstrated between activity in the anterior subdivision of the mid-cingulate cortex and the experience of more-intense states of negative affect^32^, including fear^33^. The posterior mid-cingulate cortex may play a more specific role in threat appraisal and risk assessment by approaching the threat^34^. Our significant mid-cingulate cluster encompassed both anterior mid-cingulate cortex and posterior mid-cingulate cortex, suggesting that the impaired processing of fear in ASPD + P may be related to deficits in both responsivity to intensity and threat appraisal. Relative functional deficits in these key areas of the fear processing network in ASPD + P is in keeping with a model whereby impairment in the ability to recognize and integrate distress cues (such as fear in others) predisposes such individuals to especially pronounced aggressive behavior^15^.

Our findings on the effect of oxytocin demonstrate for the first time, to our knowledge, that neural processing abnormalities in a robustly classified group of men with ASPD + P may be modified by neurochemical intervention. Oxytocin resulted in increased modulation by fearful expression intensity in ASPD + P in left anterior insula and bilateral mid-cingulate cortex. These effects resulted in the baseline differences between ASPD + P and ASPD – P in the implicit processing of others’ fear being abolished. The enhancement of fear-associated activity in these regions suggests that the fearful faces are accorded increased salience under the influence of oxytocin, with potential ‘downstream’ behavioral impacts^35,36^. The observed short-term normalization in the empathic processing of others’ distress should prompt further investigation into the neurochemical modulation of the social cognitive abnormalities in this disorder that has such a profound personal and societal impact.

Some caveats should be noted in relation to our fMRI findings. First, the finding of reduced anterior insula reactivity under placebo in ASPD + P compared with ASPD – P in the right insula did not survive strict correction for multiple comparisons, and this finding therefore requires replication in a larger sample. Second, although our baseline finding was in the right insula, our finding of increased modulation by fearful expression intensity in ASPD + P following administration of oxytocin was observed in the left insula. Inconsistencies in findings regarding hemispheric laterality of effects of oxytocin in fMRI studies^28^ have been discussed in depth elsewhere, and as a result, our finding of a unilateral effect of oxytocin should be interpreted with some caution. Third, our findings in mid-cingulate cortex contained white matter. In fMRI analyses that have low spatial/anatomical resolution and a high degree of spatial smoothing, some degree of gray matter activation being localized within ‘white matter’ as represented by sharply defined high-resolution templates is relatively common and not necessarily a sign of noise. The shape of the cingulate cortex (which loops around the surrounding white matter) makes it particularly prone to this. Although we have taken several steps to remove noisy signal arising from motion that would result in spurious activations within white matter (Supplementary Information section 3), it is possible that our findings are affected by noise, for example, due to motion.

Furthermore, while there were significant differences between ASPD + P and ASPD – P at baseline (placebo) in insula and mid-cingulate cortex, there were not significant differences between either group and NO, although the pattern of parameter estimates in the mid-cingulate cortex and insula (ASPD – P activity > NO activity > ASPD + P activity) were in the expected direction. As such, we cannot be certain that either antisocial group showed significantly atypical face responsiveness compared with NO. However, it is important to note that the ASPD + P group showed a significantly greater response to oxytocin than did the ASPD – P group within mid-cingulate cortex and left insula; that is, oxytocin selectively increased the response within this these regions to increasing fearful facial expression intensity. Moreover, previous fMRI studies in youth with conduct disorder ± callous-unemotional traits^37^ and adults with ASPD ± P^10^ have demonstrated a similar pattern to that in our study: significant differences between the two antisocial groups but not between the antisocial groups and a normal control group. This likely reflects the use of underpowered samples in a difficult-to-recruit clinical population^38–41^. This also applies to the lack of significant findings in the amygdala in between- and within-group analyses, in both placebo and oxytocin conditions (see Supplementary Information for a further consideration of this negative finding). These factors suggest caution is required in interpretation of both the positive and negative findings in our study and further emphasize the importance of larger-scale collaborative projects to address such issues.

Some other limitations should also be noted. While both ASPD groups had similar lifetime histories of substance misuse, there were some differences in substance misuse measured on the day of scanning (for example, more of the ASPD + P group had recently consumed cocaine than had the ASPD – P group). However, such group differences were carefully controlled for in the imaging analyses, and the observed group differences at baseline and in response to oxytocin cannot be attributed simply to such differences. Finally, while our study benefited from use of a task measuring implicit responding to facial expressions of fear (see Supplementary Information for further discussion), we did not examine any potential impact on observable behaviors. The potential clinical significance of our findings therefore requires further specification.

This study also had important strengths. First, this study in a carefully characterized group of participants with ASPD established differences between antisocial groups in adulthood in the implicit processing of fearful facial expressions, a central aspect of empathic responding. Second, this study investigated the neural effects of oxytocin in this group, achieved using a randomized, placebo-controlled method. Further, diagnoses and Psychopathy Checklist–revised (PCL-R) ratings were made by trained clinicians, with use of official criminal records to help classify participants.

In conclusion, we have demonstrated that the implicit processing of fearful facial emotion expressions significantly differs between violent antisocial male offenders with and without psychopathy. Oxytocin abolished these group differences in the mid-cingulate cortex. This represents evidence of neurochemical modulation of the empathic processing of others’ distress in psychopathy. Neurochemical modulation of core deficits in the condition could have profound implications for treatment of this complex disorder.

## Methods

### Participants and assessment

Between September 2017 and March 2020, we enrolled 58 men, aged 20 to 58 years, with an IQ greater than 70 as defined by the Wechsler Abbreviated Scale of Intelligence^41^. Offenders with convictions for violent crimes (murder, rape, attempted murder, grievous and actual bodily harm) who met *Diagnostic and Statistical Manual of Mental Disorders* 5th Edition (DSM-5) criteria for antisocial personality disorder (Structured Clinical Interview for DSM-5 Personality Disorders (SCID-5 PD^42^) were recruited via the National Probation Service of England and Wales and local forensic personality disorder services. Healthy non-offenders were recruited from the general population using online advertisements and fliers in job centers and local recreational centers. Offenders and controls were recruited in parallel and interlaced in the study protocol sessions outlined in the following. All participants completed diagnostic (Structured Clinical Interview for DSM-5 Personality Disorders^42^) and PCL-R^43^ interviews and authorized access to their criminal records. A cross-cultural validation study^44^ of the PCL-R demonstrated that cut-off scores for psychopathy in men vary between North America (30 out of a possible 40 points) and Europe (25 out of a possible 40 points). In line with previous research in UK samples^9,10^, we used a score of 25 as the threshold for psychopathy in this English population. We calculated total, factor 1 and factor 2 PCL-R scores for all participants. Factor 1 scores are a total of facet 1 (interpersonal traits, such as pathological lying) plus facet 2 traits (affective traits, such as lack of empathy), while factor 2 scores are a total of facet 3 (antisocial lifestyle traits, such as impulsivity) plus facet 4 traits (overt antisocial behaviors, such as criminal versatility). Exclusion criteria were history of major mental disorder (bipolar 1, bipolar 2, major depression or psychotic disorders) or self-reported neurological disorder, head injury resulting in loss of consciousness for 1 h or longer, severe visual or hearing impairment or contraindication to MRI.

After receiving a complete description of the study, all participants provided written consent. Ethical approval was obtained from the national UK research authority (National Health Service Health Research Authority Research and Ethics Committee, project number 15/LO/1083). All assessments were conducted by an experienced forensic psychiatrist (J.T.). Participants completed the Reactive–Proactive Aggression Questionnaire (RPQ)^45^. On the day of each MRI scan, participants provided a urine sample to assess for substance misuse. Following psychometric assessments, only participants who attended for two MRI sessions were included in the analyses.

The three groups did not differ significantly except for years of education and PCL-R total and facet scores (Table 2). The offenders with ASPD + P had significantly higher proactive, reactive and total aggression scores than offenders with ASPD – P. Cronbach’s alpha for internal consistency of items was 0.79 for PCL-R and 0.91 for RPQ (see Supplementary Tables 1 and 2 for inter-item correlations). The offenders with ASPD + P also had a significantly higher rate of comorbid Cluster A personality disorder diagnosis compared with NO. Offender groups (with and without psychopathy) had a significantly higher rate of comorbid Cluster B personality disorder diagnosis compared with NO. Both offender groups also had a significantly higher rate of lifetime substance misuse disorders than did NO; however, the proportion of offenders with and without psychopathy with lifetime substance-use disorders did not differ. Urinary drug screening on the day of scanning revealed some significant differences in active illicit substance misuse (Supplementary Table 1); therefore, this was included as a covariate in fMRI analysis.

**Table 2:** Demographic and clinical characteristics

	Group			Group comparison		Post hoc tests (*P* values)		
Demographic/clinical characteristic	NO (*n* = 24)	ASPD – P (*n* = 15)	ASPD + P (*n* = 19)	Statistic	*P* value	Control versus ASPD – P	Control versus ASPD + P	ASPD – P versus ASPD + P
**Age (years)**	37.6 (10.3)	40.9 (9.6)	38.7 (9.1)	0.52	0.59	0.92	1.0	1.0
**Ethnicity** **(non-white)**	7 (29.1%)	4 (26.6%)	5 (26.3%)	0.13	1.0	1.0	1.0	1.0
**IQ**	100.9 (12.6)	97.6 (16.7)	91.8 (11.3)	2.31	0.11	1.0	0.11	0.7
**Duration of education (years)**	13.9 (3.2)	10.8 (2.2)	10.0 (2.0)	13.07	<0.001**	0.002**	<0.001**	1.0
**Age at first violent conviction**	n/a	20.6 (6.2)	18.5 (5.5)	0.97	0.34	n/a		
**Number of violent convictions**	n/a	3.7 (3.1)	4.7 (2.9)	−0.87	0.39			
**RPQ Reactive Aggression**	6.0 (4.0)^a^	12.4 (4.8)^a^	17.2 (4.5)^a^	29.28	<0.001**	<0.001**	<0.001**	0.013*
**RPQ Proactive Aggression**	1.0 (1.3)^a^	7.8 (6.3)^a^	14.2 (6.2)^a^	33.71	<0.001**	0.003	<0.001**	0.016*
**RPQ Total Aggression**	6.7 (5.0)^a^	20.2 (10.8)^a^	31.4 (10.2)^a^	37.61	<0.001**	0.001	<0.001**	0.012*
**PCL-R Facet 1 (Interpersonal)**	0.75 (0.98)	2.02 (1.64)	4.42 (1.86)	32.25	<0.001**	0.038**	0.001**	0.001**
**PCL-R Facet 2 (Affective)**	0.75 (0.94)	3.13 (1.64)	5.42 (1.95)	50.40	<0.001**	0.001**	<0.001**	0.001**
**PCL-R Facet 3 (Lifestyle)**	1.16 (1.63)	5.46 (1.68)	7.52 (1.17)	99.15	<0.001**	<0.001**	<0.001**	0.001**
**PCL-R Facet 4 (Antisocial)**	0.91 (1.72)	6.33 (2.16)	8.63 (1.11)	117.96	<0.001**	<0.001**	<0.001**	<0.001**
**PCL-R Total**	2.70 (2.74)	18.41 (3.49)	28.84 (3.14)	391.56	<0.001**	<0.001**	<0.001**	<0.001**
**Personality disorder other than ASPD (%)**								
Cluster A	0	6.7	31.5	12.97	0.005**	0.38	0.0045**	0.10
Cluster B	0	20	36.8	10.19	<0.006**	0.016*	0.0016**	0.46
Cluster C	0	13.3	5.2	3.34	0.19	0.57	0.33	0.44
**Lifetime substance-use disorder (%)**	8.3	33.3	21	3.83	0.15	0.047*	0.23	0.42

Group data are mean (s.d.) unless otherwise stated. ’Statistic’ refers to *F*_(2,55)_ for continuous variables in three-group analyses; chi-squared/Fisher’s exact test for categorical data. All statistical tests were two-sided.

^a^Not all participants completed the RPQ: NO, *n* = 21; ASPD – P, *n* = 12; ASPD + P, *n* = 15.

*Statistically significant at *P* < 0.05.

**Statistically significant at *P* < 0.01.

### Study design and procedures

In a double-blind, placebo-controlled, randomized crossover design, participants self-administered, under instruction from the researcher, 40 IU of IN-OT (Syntocinon; Novartis) or placebo (identical composition to Syntocinon except for the omission of oxytocin). Participants began the morphed faces task within 25–30 min of administration. The oxytocin dose used was the highest clinically applicable safe dose administered to human volunteers, in keeping with a protocol that demonstrated significant neural activation over a period of 25–78 min with this dose^46^. Further discussion about the dose and timing of intranasal oxytocin, and mechanism for delivery to the brain, is included in Supplementary Information section 1. At a second session (occurring between 3 and 28 days later), participants completed the fMRI task again under the alternative treatment condition. Participants were instructed to avoid food, drinks (except water) and nicotine two hours before starting the experiment. Participants completed the Morphed Faces task (Supplementary Information section 1). During the task, participants were asked to indicate the sex of each face with a left–right button press using the index and middle finger of their right hand during a single run, which lasted 9 min 56 s. Full description of the Morphed Faces task, together with information on data quality control and motion parameters, is available in Supplementary Information section 1. No participant reported adverse effects following the intranasal spray.

This trial was registered at ClinicalTrials.gov (ID NCT05383300). CONSORT guidelines were adhered to, and the CONSORT statement is available in Supplementary Information. Allocation of oxytocin or placebo was randomized in advance with randomization generator software that used permuted blocks of six (http://www.randomizer.org). Intranasal oxytocin spray (Syntocinon; Novartis) and placebo were provided and administered by Maudsley Pharmacy. The active intranasal oxytocin spray was an identical formulation to the oxytocin spray, except for no active agent. A kit was allocated to each participant. Bottles for oxytocin and placebo were identical, except randomly marked A or B. Each participant was first allocated spray ‘A’ and then spray ‘B’ on the subsequent visit. On the day of collection, only pharmacy staff knew which spray had been provided, and this was recorded in their records. Both the researcher and the participant were blinded to the formulation of the spray being used. At the end of the experiment, researchers were unblinded following instruction to Maudsley Pharmacy from the study principal investigator.

The target sample size was based on a previous computational study^47^. This showed that at thresholds that approach those used after correcting for multiple comparisons, about 24 participants are required to achieve 80% power at the single-voxel level for typical activations (an 80% true positive rate) in fMRI studies.

### General linear model analysis of behavioral data

For the Morphed Faces task, means were first calculated across the whole sample for both accuracy and reaction time in rating the gender of the faces displayed. To investigate the effect of oxytocin and its interaction with other variables, for both accuracy and response latency data, a three-group (NO, ASPD – P and ASPD + P) by two-condition (oxytocin and placebo) by four-intensity (40%, 60%, 80% and 100% of fearful facial expression) repeated-measures analysis of variance was conducted. Post hoc repeated-measures analysis of variance was performed for ASPD – P versus ASPD + P. Statistical Package for the Social Sciences version 25.0 was used. A threshold for significance of *P* < 0.05 was set for all tests.

### Primary outcome measure and MRI processing

Whole-brain BOLD fMRI data were acquired using a 3.0 Tesla General Electric magnetic resonance scanner. The principle outcome measure was a regressor for modulation of neural activity (BOLD responsivity) by intensity of fearful expression. Specific MRI parameters, and full details of preprocessing and individual-level analyses and data quality control, are available in Supplementary Information sections 3 and 4.

### MRI data-group analysis

Following preprocessing steps, modulated emotion data were entered into a three-group (NO, ASPD – P and ASPD + P) by two-condition (placebo and oxytocin) 3dMVM (analysis of variance-style computations) model. Within this framework, general linear tests were coded to assess differential effects of the drug between the groups. Post hoc *t* tests were conducted to decompose these interactions by examining between- and within-group effects. Correction for multiple comparisons was performed using a spatial clustering operation in the AFNI (Analysis of Functional NeuroImages) 3dClustSim utilizing the autocorrelation function (-acf) with 10,000 Monte Carlo simulations for the whole-brain analysis. Spatial autocorrelation was estimated from residuals from the individual-level general linear models. The initial threshold was set at *P* = 0.005. As outlined in the preceding, bilateral amygdala, anterior insula and mid-cingulate cortex were selected a priori for ROI analysis. Small-volume corrections, calculated using an anatomically defined mask (TT_Daemon, a Talaraich atlas from AFNI), yielded thresholds of *k* = 13 for anterior/mid-cingulate cortex, *k* = 8 for anterior insula and *k* = 2 for amygdala at an initial significance threshold of 0.005 (multiple comparison corrected *P* < 0.05).

### Reporting summary

Further information on research design is available in the Nature Portfolio Reporting Summary linked to this article.

### Supplementary information

Supplementary Information Supplementary Materials

Reporting Summary

## Data Availability

All data are available from the authors upon reasonable request.

## Code availability

All neuroimaging data were processed using AFNI code (available at https://afni.nimh.nih.gov/download).
