# Supplementary material for: Oxytocin normalizes the implicit processing of fearful faces in psychopathy: a randomized crossover study using fMRI
Source: Nat Ment Health. 2023 May 25;1(6):420–7. doi: 10.1038/s44220-023-00067-3 (PMC11041724; doi:10.1038/s44220-023-00067-3)
Supplement: Supplementary file 1 — Supplementary Materials [file 44220_2023_67_MOESM1_ESM.pdf]

nature mental health

Article

https://doi.org/10.1038/s44220-023-00067-3

Oxytocin normalizes the implicit processing of fearful faces in psychopathy: a randomized crossover study using fMRI

In the format provided by the authors and unedited

S1: Further considerations

A. Oxytocin- mechanism of delivery to brain

As oxytocin is a relatively large peptide, it does not readily cross the blood-brain barrier when given intravenously [1]. Hence, in experimental studies in humans, oxytocin has been delivered by the intranasal route. This is a convenient and safe mode of delivering the hormone, and has been used in this way for decades, with minimal problems: oxytocin produces no detectable subjective changes in recipients, produces no reliable side-effects, and is not associated with adverse outcomes when delivered in doses of 18-40 I.U. for short term use in controlled research settings [2]. Intranasal oxytocin increases oxytocin levels in the CNS in animals [3,4] and humans [5], and a considerable volume of research has shown that that it exert effects on brain as measured by fMRI ([6,7]).

However, the mechanism by which intranasally-delivered oxytocin exerts its effects on brain remains is poorly understood and remains a point of contention [1,8]. The clearest model of this mechanism has been outlined by Quintana et al [9,10]. This proposes that intranasally delivered oxytocin, absorbed through the nasal mucosa, can reach the brain via cranial nerves: the olfactory nerve via olfactory sensory neurons located in the mucous layer, and the trigeminal nerve via trigeminal ganglion cell fibers, which are also located close to the surface of the nasal cavity. The model also asserts that intranasally-delivered oxytocin may also exert effects on CNS after entering the systemic circulation pathways via the nasal mucosa. First, it is absorbed into systemic circulation via blood capillaries located underneath the membrane of the nasal cavity. It may then: i) cross the blood-brain barrier in very small amounts- as has been demonstrated in rodents [11] and/or ii) exert effects through afferent feedback mechanisms to the CNS from receptors within peripheral organs [9]. A subsequent study comparing intranasal and intravenous delivery showed that oxytocin dampened amygdala activation in response to emotional faces in the intranasal condition only [12]. However, a more recent ASL study supports a model of effects via the systemic circulation only. This showed that both intranasally and intravenously delivered oxytocin exerted similar effects on cerebral blood flow in the oxytocinergic network (as described above) [13]. At present, a consensus on the precise mechanism of intranasally-delivered oxytocin on the CNS remains elusive.

B. Oxytocin- dose and timing

At the outset of this study, while the basic physiology of oxytocin was established [1] and a theory for mechanism of action had emerged [14], questions remained about the optimal way to manipulate the oxytocin system experimentally. Firstly, there was no consensus on optimal dose, with a range of doses being commonly used- from 8 I.U. to 40 I.U. [15]. Further, whether any dose effect was linear or more complicated (e.g. inverted-U-shaped) had not been established [16]. Also, there were several unresolved issues regarding nasal spray formulation, such as nasal spray viscosity, liposolubility, and ionisation (and these continue to exist [10]). Finally, a standardised method of delivering oxytocin intranasally was not available- i.e., different studies used different types of spray, and different timing of delivery of dose. In the context of

these uncertainties, I chose to use 40 I.U., the highest clinically applicable safe dose administered to human volunteers, and followed a local protocol developed by Paloyelis et al [17], which had recently shown successful activation of the oxytocinergic network.

Some studies have since investigated outstanding issues. Firstly, several studies have investigated the optimal dose of oxytocin. One fMRI study using a facial emotion paradigm in 116 healthy men compared both varying doses (12, 24, and 48 I.U.) and dose latencies (15–40, 45–70, and 75–100 minutes) of oxytocin in order to identify the most robust effects on amygdala reactivity [18]. Effects were most prominent with the 24 I.U. dose and at a time window between 45 and 70 minutes after administration (though neural effects were also evident at the 48 I.U. dose). However, two randomised fMRI studies in healthy subjects by Quintana et al suggested that a lower dose of oxytocin- 8 I.U.- was more effective [12,14]. Notably, these studies delivered oxytocin using a breath-powered device, which is thought to overcome the barrier to delivery posed by the nasal valve [19]. A further recent study using a dose-response design (9, 18 and 36 IU), demonstrated that intranasal oxytocin-induced changes in local regional cerebral blood flow (rCBF) in the amygdala at rest, and in the covariance between rCBF in the amygdala and other key hubs of the brain oxytocin system, follow a dose-response curve with maximal effects for lower doses [20].

In summary, while doses of 40 I.U. or similar have shown consistent effects on neural markers, the optimal formulation, timing, and dosage for intranasal oxytocin in clinical and experimental neuropsychiatric research remains unknown, and is an ongoing challenge for future work in this area (see [10] for an up-to-date review).

C. Morphed Faces Task- full details

Participants were instructed to lie as still as possible during the entire task. They were provided with a small keypad, and asked to place their index finger on button 1 and their middle finger on button 2. They were presented, on a small screen within the scanner, with images of male and female faces expressing fearful and happy expressions. They were asked to identify the sex of the face presented, by pressing button 1 for female and 2 for male. All images were of Caucasian adults (50% female) drawn from well-validated images in the Pictures of Facial Affect Series (Ekman, 1976). To allow for analysis of parametric modulation, photos displaying each target emotion were morphed with a photo of the same face displaying a neutral expression in 4 different gradients (40%, 60%, 80%, 100% of the target emotion) to produce a total of 32 unique images (4 individuals (2 men, 2 women) x 2 emotions (fearful, happy) x 4 intensities). Images were rapidly presented in a series of 50ms frames. Stimulus presentations were followed by a fixation point, which was on screen for a jittered duration of 1250-4250ms. Each subject was presented with a total of 80 fearful and 80 happy expressions, with a total task duration of 9 minutes, 56 seconds. Neural responsivity to facial emotions is typically investigated using fMRI and two-dimensional images of facial emotions. The paradigm used may be explicit, requiring participants to identify the emotion displayed (for an example in antisocial populations see (Contreras-Rodriguez, Pujol et al. 2014)) . However, one potential limitation of these paradigms in studies on individuals with ASPD+/-P is their reliance on the veracity of the subjects' responses. This is particularly relevant to research in ASPD+/-P, as the tendency to lie and manipulate are core features of ASPD+P, and may also be present to some degree in ASPD-P. Such

traits may extend into the experimental domain, leading to spurious findings, for example if subjects choose not to co-operate with the task, or provide wilfully incorrect answers. In contrast, implicit tasks of facial emotion processing seek to eliminate the potential for behavioural responses that do not give a true reflection of performance. These tasks are designed to focus subjects' attention on an unrelated process, for example, identifying the biological sex of the face shown, while measuring brain responsivity on fMRI (or electrophysiological measures) as the outcome measure. A further consideration is the intensity of facial emotional expressions, which has been shown to affect their neural processing in studies in healthy subjects (Lin, Mueller-Bardorff et al. 2016, Wang, Yu et al. 2017).

The Morphed Faces task enables the exploration of modulated intensities of fearful facial expressions, and has been previously utilized in a study of conduct disordered children [21]. The use of morphed-face stimuli carries particular advantages. At the behavioral level, use of subtly varying morphs within an emotion class increases stimulus novelty, a factor previously shown to influence brain response to emotional faces [22]. Increasing variability in emotion displays aims to reduce the habituation associated with repeated viewing of identical face-emotions [22,23]. At the neural level, use of a gradient of intensity overcomes two limitations of prototypical '100%' face-emotion displays: correlation of the degree of neural activation with the intensity of emotion expressed [24,25] and differential engagement of components of face responsive networks, including limbic regions, with increasing emotional intensity [26]. Use of morphed-face stimuli allows modelling of linear changes along a continuum and thus examination of a "dose-response" curve of neural activity changes with increases in facial emotion [23,27].

D. Lack of findings in amygdala

The lack of significant findings in the amygdala was a somewhat surprising finding. A large-scale 2009 meta-analysis suggested that the amygdala plays an important role in neural processing of fearful faces [28]. In antisocial populations, differential amygdala responsivity has been thought to underpin behavioural differences in responsivity to distress cues in CD+CU (vs CD-CU) and ASPD+P (vs ASPD-P). For instance, youth with conduct problems and high CU traits demonstrated reduced amygdala responses to fearful expressions compared to those with low CU traits [29-32], while youth with CD+CU [21,33] and adults with ASPD+P [34] demonstrate reduced amygdala responses to fearful expressions compared to healthy controls. In contrast, youth with conduct problems and low CU traits (similar to CD-CU) showed increased amygdala responses to fearful faces (compared to youth with no conduct problems [35]), and individuals with heightened reactive aggression (similar to ASPD-P) have been shown to have amygdala hyperactivity in response to facial expressions of fear [36]. However, changes in amygdala reactivity may be difficult to detect due to the relatively small size of the region [37,38] and stimulus-correlated signal fluctuation in nearby veins [39]. A recent systematic review of neuroimaging studies in antisocial populations has also cast doubt on the prominence of the role of the amygdala [40]. Specifically, this study revealed a high proportion of null findings, a disproportionate number of positive findings from low powered studies, and peak coordinates of reduced amygdala activity not primarily falling within the anatomical bounds of the amygdala. Recommendations for future work included rigorous labeling of significant clusters of voxels, large-scale studies for adequate power, and a shift in focus to neural networks, as opposed to discrete regions.

S2~~3~~: Full details of preprocessing and individual level analyses

Functional MRI data were preprocessed and analyzed using Analysis of Functional NeuroImages (AFNI) software [41]. Data from the first five repetitions were collected prior to magnetization equilibrium and were discarded. FMRI data were despiked, and volumes were censored if meeting a motion threshold of >1mm or if 10% of voxels within a volume met outlier criteria as determined by 3dTOutcount (none did). The anatomical scan for each participant was registered to the Talairach and Tournoux atlas [42] and each participant's functional EPI data were registered to their Talairach anatomical scan in AFNI. Functional images were motion corrected and spatially smoothed with a 6-mm full width half maximum Gaussian kernel. The data then underwent time series normalization and these results were multiplied by 100 for each voxel. The resultant regression coefficients are therefore representative of a percentage of signal change from the mean.

At the single subject level, regressors depicting each of the response types and nuisance motion regressors were then created by convolving the train of stimulus events and realignment parameters with a gamma-variate haemodynamic response function to account for the slow haemodynamic response. Regressors of interest included i) fear; ii) its parametric modulation by intensity; iii) happiness; iv) its parametric modulation by intensity. Linear regression modelling was then performed using the regressors described above plus regressors to model a first order baseline drift function. This produced a beta coefficient and its associated t-statistic for each voxel and each regressor. The modulated regressors were then taken forward for the group level analyses.

The parametric modulation of neural responses by fearful facial emotion intensity (regressor 2 above) data were then entered into a 3 Group (NO, ASPD-P, ASPD+P) vs 2 Condition (placebo, oxytocin) 3dMVM (ANOVA style computations) model for fearful expressions. As there were significant group differences in illicit drug use, established by urinary drug screening on the day (see Supplementary Table 1), and there was a feasible mechanistic basis for a consequent effect on neural responsivity, this was incorporated as a covariate. This model provides outputs for the overall effects of task, group, and condition. Within this framework, general linear tests were coded to assess differential effects of condition (oxytocin or placebo) between the groups. To investigate responsivity to the task itself, the overall intercept (F) was used. Post hoc pairwise comparisons were conducted to decompose these interactions by examining between and within group effects. Correction for multiple comparisons was performed using a spatial clustering operation in AFNI's 3dClustSim utilizing the autocorrelation function (-acf) with 10,000 Monte Carlo simulations for the whole-brain analysis. Spatial autocorrelation was estimated from residuals from the individual-level GLMs. The initial threshold was set at $p = 0.001$. This process yielded an extant threshold of $k = 22$ voxels for the whole brain (multiple comparison corrected; $p < 0.05$).

S34: Data Quality Control

Volumes were censored if there was 1mm motion across adjacent volumes. No participant in the final sample for the current study had >5% censored volumes. Two group (NO vs All ASPD) by two condition (placebo vs oxytocin) repeated measures ANOVAs (with post-hoc ASPD-P vs ASPD+P analysis) were carried out for five motion parameters: TRs above threshold, average motion per TR, maximum displacement during scanning, outcount, and maximum F. These revealed no significant group or condition differences (multiple comparison corrected). Data was also manually checked for quality using two main steps. Firstly, the alignment of each scan was checked by visually inspecting coronal, sagittal and axial images. Secondly, the functional (EPI) imaging sequences were inspected for each scan to screen out highly abnormal activation patterns which were likely to represent poor quality data. Following such quality checks, one subject was excluded due to excess subject head movement during the task.

S45. Recruitment and participation

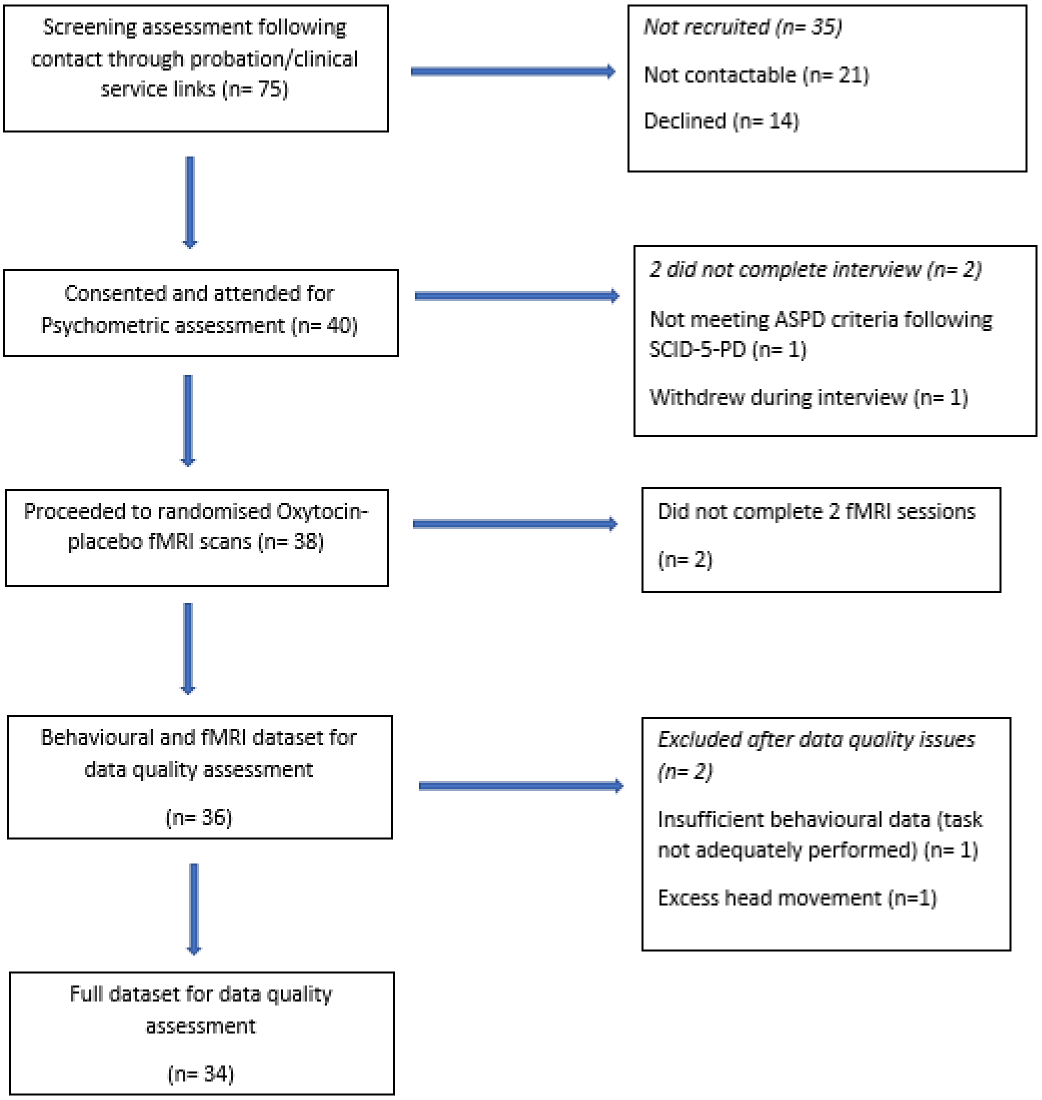

Figure S1. Flowchart of recruitment and participation of offenders with antisocial personality disorder with or without psychopathy (ASPD+/-P).

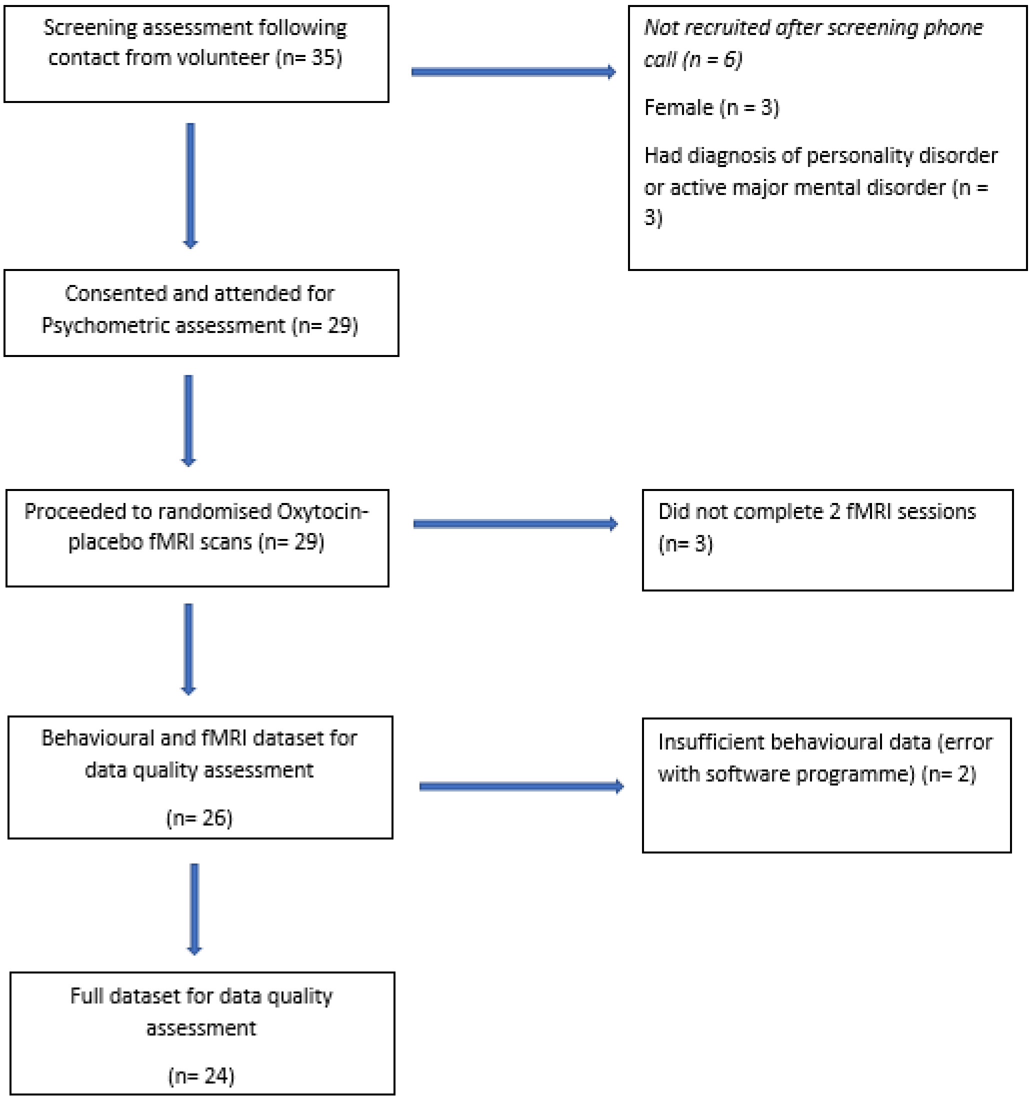

Figure S2. Flowchart of recruitment and participation of non-offending health controls (NO).

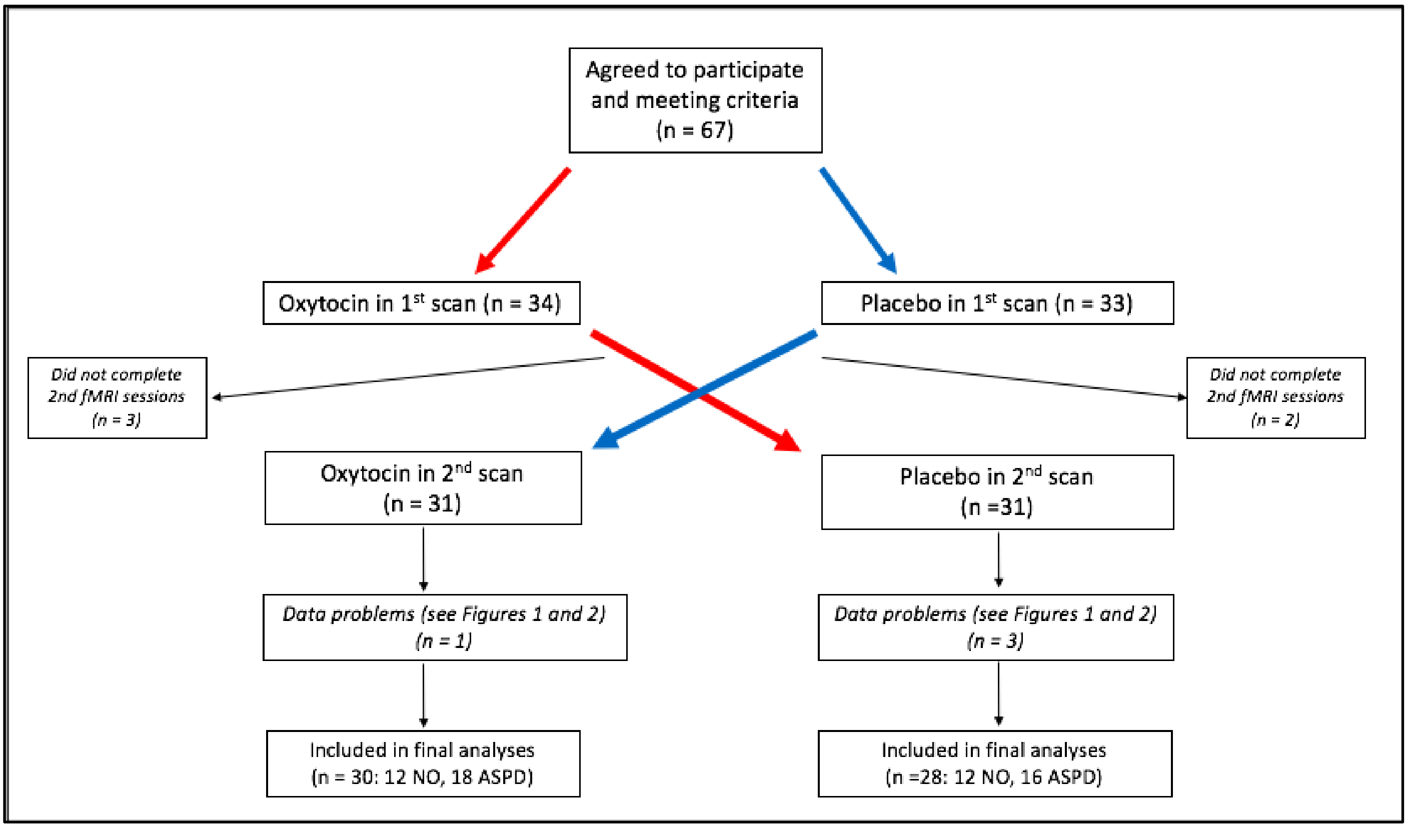

Figure S3. Flowchart of crossover study design.

S5~~6~~. Inter-item correlations and internal consistency of PCL-R and RPQ

	Facet 1	*Facet 2*	*Facet 3*	*Facet 4*	***Factor 1***	***Factor 2***
Facet 1	-	0.784*	0.786*	0.781*	**0.93***	**0.832***
Facet 2	0.784*	-	0.82*	0.883*	**0.76***	**0.911***
Facet 3	0.786*	0.82*	-	0.881*	**0.71***	**0.904***
Facet 4	0.781*	0.883*	0.881*	-	**0.735***	**0.869***
Factor 1	**0.93***	**0.76***	**0.71***	**0.735***	**-**	**0.792***
Factor 2	**0.823***	**0.911***	**0.904***	**0.869***	**0.792***	**-**

Table S1. Correlations between PCL-R facets and factors. Cronbach's alpha for four facets was 0.79. *significant at p<0.001

	Reactive aggression	*Proactive aggression*	***Total Aggression***
Reactive aggression	-	0.855*	**0.952***
Proactive aggression	0.835*	-	**0.963***
Total Aggression	**0.952***	**0.963***	**-**

Table S2. Correlations between Reactive Proactive aggression Questionnaire (RPQ). Cronbach's alpha for the two subscales was 0.905. *significant at p<0.001

S67: Supplementary Results

Main effect of task: parametric modulation of neural responses by fearful facial emotion intensity in all subjects

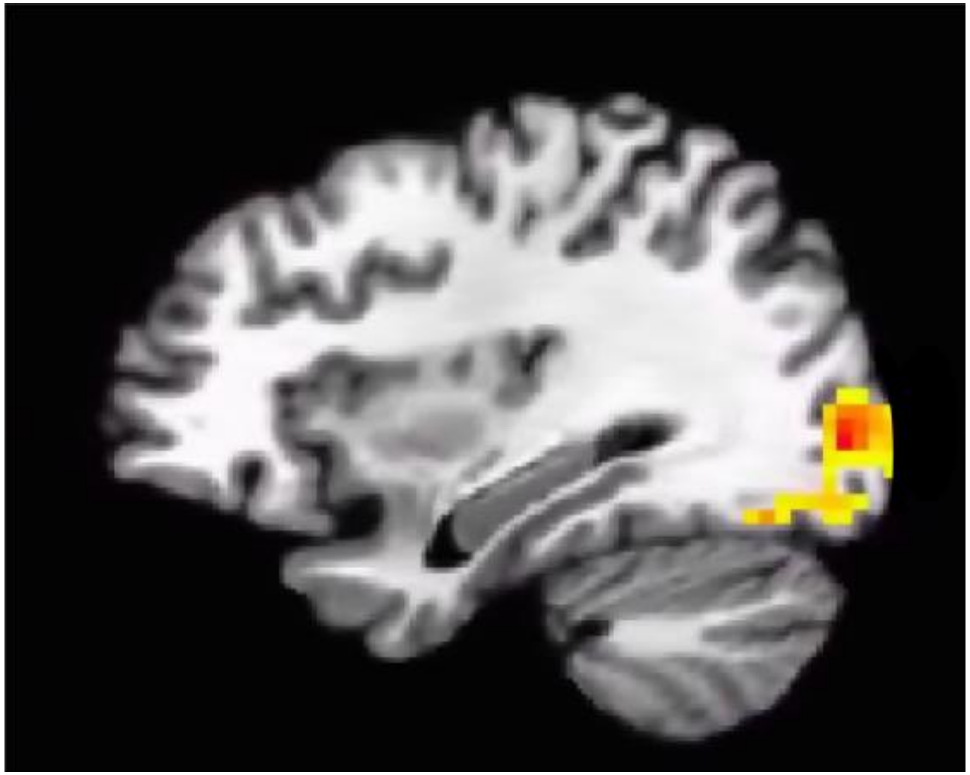

Figure S3. Increased activity in occipital cortex and fusiform cortex, significant at p < 0.001 (see table S1).

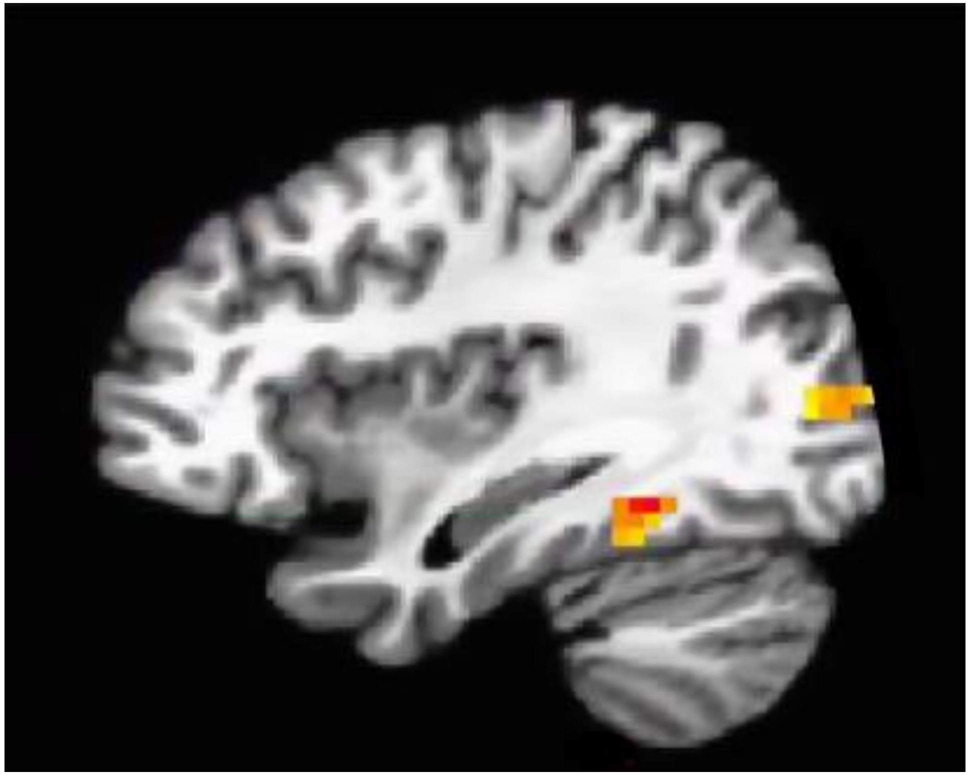

Figure S4. Increased activity in occipital cortex and fusiform cortex, significant at p < 0.001. Only the occipital cortex finding survived clusterwise correction for multiple comparisons (see table S1).

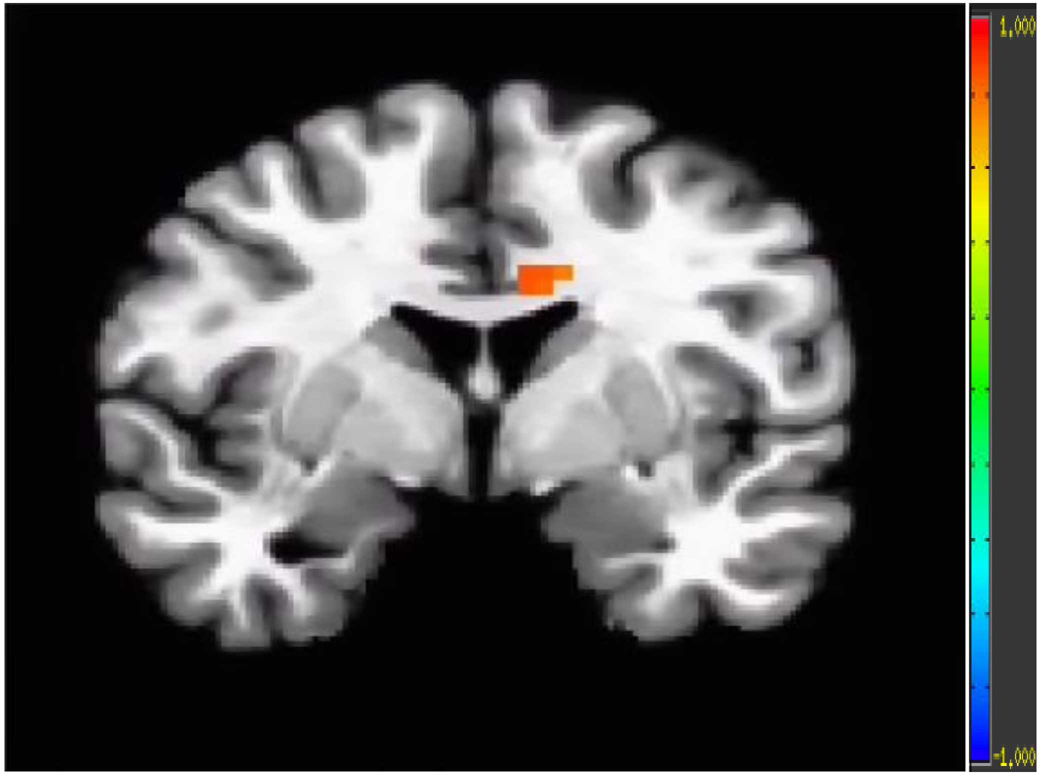

Figure S5. Significant group (ASPD+P v ASPD-P) x condition (placebo vs oxytocin) interaction effect in left midcingulate cortex during fear processing. Color bar represents t statistic.

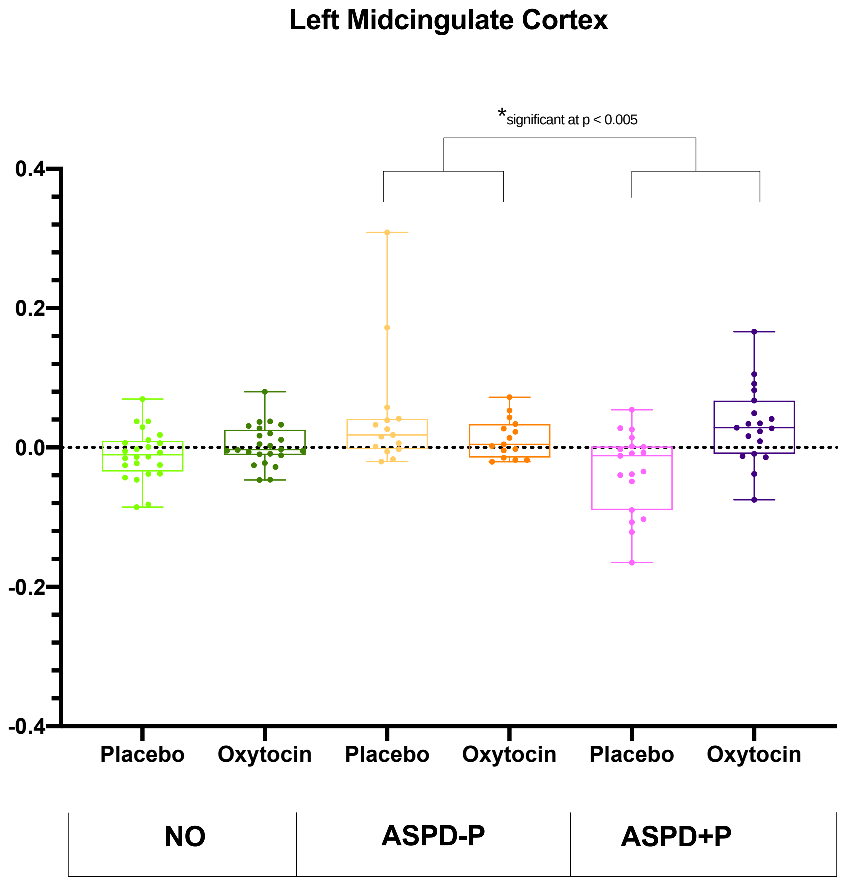

Figure S6. Individual beta values for group (ASPD+P v ASPD-P) x condition (placebo vs oxytocin) interaction effect in left midcingulate cortex during fear processing. Individual subjects' data plotted as dots. Means are indicated by horizontal bars. Error bars represent standard deviations. NO= non-offenders ASPD-P = violent offenders with antisocial personality disorder but not psychopathy. ASPD+P= violent offenders with antisocial personality disorder and psychopathy

Region	BA	Voxels	X	Y	Z	F	p
Overall effect of task							
R middle occipital gyrus	18/19	223	-31.5	+82.5	-0.5	26.33	<0.0001
L middle occipital gyrus	17/18	49	255	885	25	15.64	0.0002
L fusiform gyrus	-	38	345	495	-155	22.63	<0.0001

Table S3. Main effect of task- parameter details
*Did not survive clusterwise correction for multiple comparisons (22 voxel threshold).

	Group			*Group comparison*		*Post Hoc Tests (p values)*		
**Demographic/ Clinical Characteristic**	**NO (n =24)**	**ASPD-P (n= 15)**	**ASPD+P (n= 19)**	**Statistic[a]**	**P value**	**Control vs ASPD-P**	**Control vs ASPD+P**	**ASPD-P vs ASPD+P**
**Any drug**	6 (25%)	4 (26.6%)	14 (73.6%)	12.16	0.002	1.0	0.002	0.014
**Cannabis**	5 (20.8%)	3 (20%)	9 (47.3%)	4.45	0.108	n/a		
**Cocaine**	2 (8.3%)	1 (6.6%)	8 (42.1%)	9.86	0.007	1.0	0.013	0.046
**Opioid**	0 (0%)	1 (6.6%)	3 (15.7)	4.11	0.128	n/a		
**Benzodiazepine**	0 (0%)	0 (0%)	4 (21%)	8.81	0.012	1.0	0.031	0.113
**Amphetamine**	0 (0%)	0 (0%)	1 (5.2%)	2.08	0.352	n/a		
**Other hallucinogenic**	1 (4.1%)	0 (0%)	0 (0%)	1.44	0.486	n/a		

Table S4. Positive illicit drug tests

References

1 Leng G, Ludwig M. Intranasal oxytocin: myths and delusions. Biological psychiatry. 2016;79(3):243-50.

2 MacDonald E, Dadds MR, Brennan JL, Williams K, Levy F, Cauchi AJ. A review of safety, side-effects and subjective reactions to intranasal oxytocin in human research. Psychoneuroendocrinology. 2011;36(8):1114-26.

3 Neumann ID, Maloumby R, Beiderbeck DI, Lukas M, Landgraf R. Increased brain and plasma oxytocin after nasal and peripheral administration in rats and mice. Psychoneuroendocrinology. 2013;38(10):1985-93.

4 Freeman SM, Samineni S, Allen PC, Stockinger D, Bales KL, Hwa GG, et al. Plasma and CSF oxytocin levels after intranasal and intravenous oxytocin in awake macaques. Psychoneuroendocrinology. 2016;66:185-94.

5 Striepens N, Kendrick KM, Hanking V, Landgraf R, Wüllner U, Maier W, et al. Elevated cerebrospinal fluid and blood concentrations of oxytocin following its intranasal administration in humans. Scientific reports. 2013;3:3440.

6 Bethlehem RA, van Honk J, Auyeung B, Baron-Cohen S. Oxytocin, brain physiology, and functional connectivity: a review of intranasal oxytocin fMRI studies. Psychoneuroendocrinology. 2013;38(7):962-74.

7 Wang D, Yan X, Li M, Ma Y. Neural substrates underlying the effects of oxytocin: a quantitative meta-analysis of pharmaco-imaging studies. Social cognitive and affective neuroscience. 2017;12(10):1565-73.

8 Bos PA, Panksepp J, Bluthé R-M, van Honk J. Acute effects of steroid hormones and neuropeptides on human social–emotional behavior: a review of single administration studies. Frontiers in neuroendocrinology. 2012;33(1):17-35.

9 Quintana DS, Alvares GA, Hickie IB, Guastella AJ. Do delivery routes of intranasally administered oxytocin account for observed effects on social cognition and behavior? A two-level model. Neuroscience & Biobehavioral Reviews. 2015;49:182-92.

10 Quintana DS, Smerud KT, Andreassen OA, Djupesland PG. Evidence for intranasal oxytocin delivery to the brain: recent advances and future perspectives. Therapeutic Delivery. 2018;9(7):515-25.

11 Mens WB, Witter A, Greidanus TBVW. Penetration of neurohypophyseal hormones from plasma into cerebrospinal fluid (CSF): half-times of disappearance of these neuropeptides from CSF. Brain research. 1983;262(1):143-49.

12 Quintana DS, Westlye LT, Alnæs D, Rustan ØG, Kaufmann T, Smerud KT, et al. Low dose intranasal oxytocin delivered with Breath Powered device dampens amygdala response to emotional stimuli: A peripheral effect-controlled within-subjects randomized dose-response fMRI trial. Psychoneuroendocrinology. 2016;69:180-88.

13 Martins D, Mazibuko N, Zelaya F, Vasilakopoulou S, Loveridge J, Oates A, et al. Do direct nose-to-brain pathways underlie intranasal oxytocin-induced changes in regional cerebral blood flow in humans? bioRxiv. 2019:563056.

14 Quintana D, Westlye LT, Rustan ØG, Tesli N, Poppy C, Smevik H, et al. Low-dose oxytocin delivered intranasally with Breath Powered device affects social-cognitive behavior: a randomized four-way crossover trial with nasal cavity dimension assessment. Translational psychiatry. 2015;5(7):e602.

15 Wigton R, Jocham Radua PA, Averbeck B, Meyer-Lindenberg A, McGuire P, Shergill SS, et al. Neurophysiological effects of acute oxytocin administration: systematic review and meta-analysis of placebo-controlled imaging studies. Journal of psychiatry & neuroscience: JPN. 2015;40(1):E1.

16 Bartz JA, Zaki J, Bolger N, Ochsner KN. Social effects of oxytocin in humans: context and person matter. Trends in cognitive sciences. 2011;15(7):301-09.

17 Paloyelis Y, Doyle OM, Zelaya FO, Maltezos S, Williams SC, Fotopoulou A, et al. A spatiotemporal profile of in vivo cerebral blood flow changes following intranasal oxytocin in humans. Biological psychiatry. 2016;79(8):693-705.

18 Spengler FB, Schultz J, Scheele D, Essel M, Maier W, Heinrichs M, et al. Kinetics and dose dependency of intranasal oxytocin effects on amygdala reactivity. Biological psychiatry. 2017;82(12):885-94.
19 Djupesland PG, Skretting A, Winderen M, Holand T. Breath actuated device improves delivery to target sites beyond the nasal valve. The Laryngoscope. 2006;116(3):466-72.
20 Martins D, Brodmann K, Veronese M, Dipasquale O, Mazibuko N, Schuschnig U, et al. "Less is more": A dose-response account of intranasal oxytocin pharmacodynamics in the human brain. Progress in Neurobiology. 2022;211:102239.
21 Marsh AA, Finger EC, Mitchell DG, Reid ME, Sims C, Kosson DS, et al. Reduced amygdala response to fearful expressions in children and adolescents with callous-unemotional traits and disruptive behavior disorders. American Journal of Psychiatry. 2008;165(6):712-20.
22 Blair KS, Geraci M, Korelitz K, Otero M, Towbin K, Ernst M, et al. The pathology of social phobia is independent of developmental changes in face processing. American Journal of Psychiatry. 2011;168(11):1202-09.
23 Thomas LA, Brotman MA, Muhrer EJ, Rosen BH, Bones BL, Reynolds RC, et al. Parametric modulation of neural activity by emotion in youth with bipolar disorder, youth with severe mood dysregulation, and healthy volunteers. Archives of General Psychiatry. 2012;69(12):1257-66.
24 Blair R, Morris JS, Frith CD, Perrett DI, Dolan RJ. Dissociable neural responses to facial expressions of sadness and anger. Brain. 1999;122(5):883-93.
25 Morris JS, Frith CD, Perrett DI, Rowland D, Young AW, Calder AJ, et al. A differential neural response in the human amygdala to fearful and happy facial expressions. Nature. 1996;383(6603):812-15.
26 Azuma R, Deeley Q, Campbell LE, Daly EM, Giampietro V, Brammer MJ, et al. An fMRI study of facial emotion processing in children and adolescents with 22q11. 2 deletion syndrome. Journal of neurodevelopmental disorders. 2015;7(1):1-16.
27 Brotman MA, Deveney CM, Thomas LA, Hinton KE, Yi JY, Pine DS, et al. Parametric modulation of neural activity during face emotion processing in unaffected youth at familial risk for bipolar disorder. Bipolar disorders. 2014;16(7):756-63.
28 Fusar-Poli P, Placentino A, Carletti F, Landi P, Allen P, Surguladze S, et al. Functional atlas of emotional faces processing: a voxel-based meta-analysis of 105 functional magnetic resonance imaging studies. Journal of psychiatry & neuroscience. 2009.
29 Viding E, Sebastian CL, Dadds MR, Lockwood PL, Cecil CA, De Brito SA, et al. Amygdala response to preattentive masked fear in children with conduct problems: the role of callous-unemotional traits. American Journal of Psychiatry. 2012;169(10):1109-16.
30 Fanti KA, Konikou K, Cohn M, Popma A, Brazil IA. Amygdala functioning during threat acquisition and extinction differentiates antisocial subtypes. Journal of neuropsychology. 2019.
31 Lozier LM, Cardinale EM, VanMeter JW, Marsh AA. Mediation of the relationship between callous-unemotional traits and proactive aggression by amygdala response to fear among children with conduct problems. JAMA psychiatry. 2014;71(6):627-36.
32 Jones AP, Laurens KR, Herba CM, Barker GJ, Viding E. Amygdala hypoactivity to fearful faces in boys with conduct problems and callous-unemotional traits. American Journal of Psychiatry. 2009;166(1):95-102.
33 White SF, Marsh AA, Fowler KA, Schechter JC, Adalio C, Pope K, et al. Reduced amygdala response in youths with disruptive behavior disorders and psychopathic traits: decreased emotional response versus increased top-down attention to nonemotional features. American Journal of Psychiatry. 2012;169(7):750-58.
34 Contreras-Rodriguez O, Pujol J, Batalla I, Harrison BJ, Bosque J, Ibern-Regas I, et al. Disrupted neural processing of emotional faces in psychopathy. Social Cognitive & Affective Neuroscience. 2014;9(4):505-12.
35 Sebastian C, McCrory E, Dadds M, Cecil C, Lockwood P, Hyde Z, et al. Neural responses to fearful eyes in children with conduct problems and varying levels of callous–unemotional traits. Psychological Medicine. 2014;44(1):99-109.

36 Coccaro EF, McCloskey MS, Fitzgerald DA, Phan KL. Amygdala and orbitofrontal reactivity to social threat in individuals with impulsive aggression. Biological psychiatry. 2007;62(2):168-78.
37 Hyde LW, Shaw DS, Murray L, Gard A, Hariri AR, Forbes EE. Dissecting the role of amygdala reactivity in antisocial behavior in a sample of young, low-income, urban men. Clinical psychological science. 2016;4(3):527-44.
38 Zald DH. The human amygdala and the emotional evaluation of sensory stimuli. Brain Research Reviews. 2003;41(1):88-123.
39 Boubela RN, Kalcher K, Huf W, Seidel E-M, Derntl B, Pezawas L, et al. fMRI measurements of amygdala activation are confounded by stimulus correlated signal fluctuation in nearby veins draining distant brain regions. Scientific reports. 2015;5:10499.
40 Deming P, Heilicher M, Koenigs M. How reliable are amygdala findings in psychopathy? A systematic review of MRI studies. Neuroscience & Biobehavioral Reviews. 2022:104875.
41 Cox RW. AFNI: software for analysis and visualization of functional magnetic resonance neuroimages. Computers and Biomedical Research. 1996;29:162-73.
42 Talairach J, Tournoux P. Co-planar stereotaxic atlas of the human brain. Thieme: Stuttgart; 1988.
