# Supplementary material for: Oxytocin normalizes the implicit processing of fearful faces in psychopathy: a randomized crossover study using fMRI
Source: Nat Ment Health. 2023 May 25;1(6):420–7. doi: 10.1038/s44220-023-00067-3 (PMC11041724; doi:10.1038/s44220-023-00067-3)
Supplement: Supplementary file 2 — Reporting Summary [file 44220_2023_67_MOESM2_ESM.pdf]

nature portfolio

Corresponding author(s): John Tully

Last updated by author(s): 240223

Reporting Summary

Nature Portfolio wishes to improve the reproducibility of the work that we publish. This form provides structure for consistency and transparency in reporting. For further information on Nature Portfolio policies, see our Editorial Policies and the Editorial Policy Checklist.

Statistics

For all statistical analyses, confirm that the following items are present in the figure legend, table legend, main text, or Methods section.

n/a	Confirmed	
☐	☒	The exact sample size (*n*) for each experimental group/condition, given as a discrete number and unit of measurement
☐	☒	A statement on whether measurements were taken from distinct samples or whether the same sample was measured repeatedly
☐	☒	The statistical test(s) used AND whether they are one- or two-sided<br>*Only common tests should be described solely by name; describe more complex techniques in the Methods section.*
☐	☒	A description of all covariates tested
☐	☒	A description of any assumptions or corrections, such as tests of normality and adjustment for multiple comparisons
☐	☒	A full description of the statistical parameters including central tendency (e.g. means) or other basic estimates (e.g. regression coefficient) AND variation (e.g. standard deviation) or associated estimates of uncertainty (e.g. confidence intervals)
☐	☒	For null hypothesis testing, the test statistic (e.g. F, t, r) with confidence intervals, effect sizes, degrees of freedom and P value noted<br>*Give P values as exact values whenever suitable.*
☒	☐	For Bayesian analysis, information on the choice of priors and Markov chain Monte Carlo settings
☒	☐	For hierarchical and complex designs, identification of the appropriate level for tests and full reporting of outcomes
☐	☒	Estimates of effect sizes (e.g. Cohen's d, Pearson's r), indicating how they were calculated

Our web collection on statistics for biologists contains articles on many of the points above.

Software and code

Policy information about availability of computer code

Data collection	Psychometric data was entered manually into SPSS and Excel spreadsheets. Neuroimaging data was stored and accessed via centralised database (NaN), at Centre of Neuroimaging Sciences, Institute of Psychiatry, Psychology and Neuroscience, Kings College London.
Data analysis	Psychoemtric analysis was done with SPSS version 25. Neuroimaging analysis was done with AFNI Version 19.0.9. All neuroimaging data was processed using AFNI code (available at https://afni.nimh.nih.gov/download).

For manuscripts utilizing custom algorithms or software that are central to the research but not yet described in published literature, software must be made available to editors and reviewers. We strongly encourage code deposition in a community repository (e.g. GitHub). See the Nature Portfolio guidelines for submitting code & software for further information.

Data

Policy information about availability of data

All manuscripts must include a data availability statement. This statement should provide the following information, where applicable:

- Accession codes, unique identifiers, or web links for publicly available datasets
- A description of any restrictions on data availability
- For clinical datasets or third party data, please ensure that the statement adheres to our policy

All data is available from the authors upon reasonable request. All neuroimaging data was processed using AFNI code (available at https://afni.nimh.nih.gov/download).

Human research participants

Policy information about studies involving human research participants and Sex and Gender in Research.

Reporting on sex and gender	Sex is specified in tables and results
Population characteristics	Yes, all data available on basic demographics, violent offending, and clinical diagnoses reported
Recruitment	Detailed in 'Methods- Participants and assessment'
Ethics oversight	Detailed in 'Methods- Participants and assessment'

Note that full information on the approval of the study protocol must also be provided in the manuscript.

Field-specific reporting

Please select the one below that is the best fit for your research. If you are not sure, read the appropriate sections before making your selection.

☐ Life sciences ☒ Behavioural & social sciences ☐ Ecological, evolutionary & environmental sciences

For a reference copy of the document with all sections, see nature.com/documents/nr-reporting-summary-flat.pdf

Behavioural & social sciences study design

All studies must disclose on these points even when the disclosure is negative.

Study description	Randomised, double-blinded, placebo-controlled, crossover study
Research sample	Adult male violent offenders with antisocial personality disorder +/- psychopathy, healthy non-offending adult male controls
Sampling strategy	Offenders recruited from probation and clinical services, controls recruited from the community by adverts
Data collection	Psychometric data collected manually by researchers and entered onto Excel and SPSS spreadsheets. MRI data automatically collected and stored on centalised database (NaN) at Centre of Neuroimaging Sciences, Institute of Psychiatry, Psychology and Neuroscience, Kings College London.
Timing	Samples recruited between September 2017 and March 2020
Data exclusions	A small number of subjects dropped out or did not generate sufficient neuroimaging data, as detailed in Supplementary Materials, Figures S1-S3.
Non-participation	n/a
Randomization	Detailed in 'Study Design and Procedures'- Allocation of oxytocin or placebo was randomised in advance with randomisation generator software that used permuted blocks of six (http://www.randomizer.org).

Reporting for specific materials, systems and methods

We require information from authors about some types of materials, experimental systems and methods used in many studies. Here, indicate whether each material, system or method listed is relevant to your study. If you are not sure if a list item applies to your research, read the appropriate section before selecting a response.

Materials & experimental systems

n/a	Involved in the study	
☒	☐	Antibodies
☒	☐	Eukaryotic cell lines
☒	☐	Palaeontology and archaeology
☒	☐	Animals and other organisms
☐	☒	Clinical data
☒	☐	Dual use research of concern

Methods

n/a	Involved in the study	
☒	☐	ChIP-seq
☒	☐	Flow cytometry
☒	☐	MRI-based neuroimaging

Clinical data

Policy information about clinical studies

All manuscripts should comply with the ICMJE guidelines for publication of clinical research and a completed CONSORT checklist must be included with all submissions.

Clinical trial registration	This trial was registered at ClinicalTrials.gov (ID NCT05383300).
Study protocol	Available at clinicaltrials.gov: https://clinicaltrials.gov/ct2/show/NCT05383300?term=NCT05383300&draw=2&rank=1
Data collection	Detailed in METHODS. Clinical data based on interviews, neuroimaging data generated from MRI scanning sessions.
Outcomes	Detailed in METHODS. For key neuroimaging outcomes measures, general linear tests were coded to assess differential effects of drug between the groups. Post hoc t-tests were conducted to decompose these interactions by examining between- and within-group effects.
